# Efficient discovery of abundant post-translational modifications and spectral pairs using peptide mass and retention time differences

**DOI:** 10.1186/1471-2105-10-S1-S50

**Published:** 2009-01-30

**Authors:** Yan Fu, Wei Jia, Zhuang Lu, Haipeng Wang, Zuofei Yuan, Hao Chi, You Li, Liyun Xiu, Wenping Wang, Chao Liu, Leheng Wang, Ruixiang Sun, Wen Gao, Xiaohong Qian, Si-Min He

**Affiliations:** 1 Institute of Computing Technology, Chinese Academy of Sciences, Beijing 100190, PR China; 2 State Key Laboratory of Proteomics-Beijing Proteome Research Center-Beijing Institute of Radiation Medicine, Beijing 102206, PR China; 3 Key Lab of Intelligent Information Processing, Chinese Academy of Sciences, Beijing 100190, PR China

## Abstract

**Background:**

Peptide identification via tandem mass spectrometry is the basic task of current proteomics research. Due to the complexity of mass spectra, the majority of mass spectra cannot be interpreted at present. The existence of unexpected or unknown protein post-translational modifications is a major reason.

**Results:**

This paper describes an efficient and sequence database-independent approach to detecting abundant post-translational modifications in high-accuracy peptide mass spectra. The approach is based on the observation that the spectra of a modified peptide and its unmodified counterpart are correlated with each other in their peptide masses and retention time. Frequently occurring peptide mass differences in a data set imply possible modifications, while small and consistent retention time differences provide orthogonal supporting evidence. We propose to use a bivariate Gaussian mixture model to discriminate modification-related spectral pairs from random ones. Due to the use of two-dimensional information, accurate modification masses and confident spectral pairs can be determined as well as the quantitative influences of modifications on peptide retention time.

**Conclusion:**

Experiments on two glycoprotein data sets demonstrate that our method can effectively detect abundant modifications and spectral pairs. By including the discovered modifications into database search or by propagating peptide assignments between paired spectra, an average of 10% more spectra are interpreted.

## Background

Identification of peptides, especially post-translationally modified peptides, using liquid chromatography coupled with tandem mass spectrometry (LC-MS/MS) is the basic task of current proteomics research [1-3]. Database search is the most widely used computational approach to peptide identification from mass spectra [4-9]. Other approaches include *de novo *sequencing [10-13] and tag-based approach [14-17]. However, due to the complexity of mass spectra, the majority (70–90%) of them cannot be interpreted at present [18]. Among many reasons for the low interpretation rate of mass spectra, unexpected or unknown peptide modifications is a major one [19,20].

Identification of modified peptides is usually conducted in a restrictive manner; that is, a set of variable modifications are specified before database search. However, there are hundreds of known natural or artificial modifications (563 entries in the Unimod [21] database up to July 28, 2008). Most of them have multiple specific sites. Therefore, it is no practical to select all the modifications for database search, since this will lead to combinatorial explosion of search space as well as increased chance of random matches. In current popular search engines, such as SEQUEST [4] and Mascot [5], no more than ten variable modification types are allowed. The problem is that in most cases we know little about which modifications occur in the protein sample and exist in the mass spectra in hand. Most of the time, oxidation on methionine is the only variable modification specified for database search. As a result, a large amount of spectra from modified peptides have not been interpreted in the past.

To address the above problem, unrestrictive approaches to modification identification have been proposed in recent years [22-25]. MS-Alignment is the first algorithm for unrestrictive identification of modifications [22,26], which aligns the experimental spectrum against the theoretical spectrum predicted from a peptide in the database in a modification-tolerant manner, just like the sequence alignment in genomics. In this way, any modifications can be identified as long as the two spectra compared present enough similarities. SPIDER formulates modification identification as a dynamic programming problem, searching for a modified peptide that minimizes the difference between the *de novo *sequenced and the database peptides [25].

Although the above approaches to unrestrictive identification of modifications are useful and attractive, they involve time-consuming database search processes or rely on good spectrum quality. If we can know the real types of modifications presented in the spectra prior to database search, the time spent will be reduced significantly. In fact, several methods have been proposed to detect modifications independently of sequence databases [19,27,28]. Due to the dynamic nature of modifications, the modified and unmodified forms of the same peptide often exist simultaneously in a protein sample. Mass spectra of modified and unmodified peptides are correlated with each other in their peptide masses, LC retention time and fragment peaks. Savitski et al. [19] proposed to use the peptide mass difference histogram constructed from paired spectra to detect modifications. However, their method builds on the complementary use of CAD and ECD fragmentation modes in the mass spectrometer, and thus is not applicable to current common proteomic experiments where only CAD or ECD is used. Potthast et al. [27] described a method called mass distance fingerprint to detect modification types from common proteomic mass spectra. In contrast to their rather complicated statistical model of mass distribution, they used the peptide mass information only, limiting the confidence of discoveries. Bandeira et al. [28] proposed to detect modification-related spectral pairs by comparing the peptide fragmentation data. Their method is applicable to both abundant and low-concentration modifications, but at the cost of computational efficiency.

This paper describes a simple yet efficient approach to detecting abundant modifications in high-accuracy peptide mass spectra using both peptide mass and retention time information. Each pair of spectra is represented by a two-dimensional (2-D) vector composed of the mass difference and the retention time difference between their precursor ions. A bivariate Gaussian mixture model is used to discriminate modification-related spectral pairs from random ones. In this way, accurate modification masses and confident spectral pairs can be obtained. We also use a peptide propagation method to assign peptides to modification spectra at given false discovery rate (FDR) without searching a database. Experiments on two glycoprotein data sets demonstrate the effectiveness of our method.

Although our method is at present unable (not designed) to find low-concentration modifications, it possesses several advantages compared to previous methods to detect modifications and spectral pairs:

Fast

Only the peptide mass and retention time information is used. Computing the spectra similarity based on peptide fragmentation data enables detection of low-concentration modifications but is very time-consuming. Clustering spectra according to the 2-D peptide mass and retention time data is demonstrated to be very fast. Abundant modifications and corresponding spectral pairs can be efficiently detected in this way as shown in Results section of the paper.

Accurate

Peptide retention time is used in addition to peptide mass. Although using peptide masses alone can also reveal modifications, retention time provides an independent source of supporting evidence. Two features are far more discriminative in detecting modification-related spectral pairs than only one feature. Therefore, modification masses and identities can be more accurately determined.

Robust

Our approach to modification and spectral pair discovery is independent of peptide fragmentation data. It is known that modified peptides often have complex fragmentation patterns. For example, phosphorylated peptides often undergo insufficient fragmentation, resulting in dominant neutral-loss precursor peaks. Peptides of cation modifications have even poorer fragmentation spectra with reduced fragment signals according to our observation. Therefore, those methods using peptide fragmentation data to measure the spectra similarity are unreliable to detect pairs of poorly fragmented spectra. The method proposed in this paper is naturally immune to this problem.

## Methods

Roughly speaking, we use two steps to look for modifications. In the first step, the peptide masses are used alone to obtain a list of candidate modification masses. In the second step, for each candidate modification mass, the retention time is added to validate the modification. Each possible pair of spectra is represented by a 2-D vector of peptide mass difference and retention time difference. Then, a bivariate Gaussian mixture model is used to differentiate modification-related spectral pairs from random ones. By doing this, the accurate modification mass values can be estimated and the influence of the modifications on retention time can be characterized. Before all of these analyses, the spectra data set is first reduced to remove redundancy, as described below.

### Removal of spectra redundancy

In tandem mass spectrometry, abundant peptides will produce many duplicate spectra, leading to data redundancy. The disadvantages of spectra redundancy in our problem lie in two folds. First, redundancy brings high computational burden. Since we are dealing with the peptide mass and retention time differences between all pairs of spectra, the size of the problem increases quadratically with the number of spectra. Second and no less important, the spectra redundancy may cause unexpected effect on the distributions of peptide mass and retention time differences. The retention time of abundant peptides may possibly distribute across a large range. This is obviously undesirable for our analysis. To remove the spectra redundancy, among all the spectra whose peptide mass values are within a small window (e.g. 5 ppm for FT instruments), only one of them is reserved as the representative spectrum and all other spectra are removed. Of course, spectra of close peptide masses may not be produced by the same peptide and some spectra may be falsely removed as redundant spectra. However, this hardly affects our analysis. We find that a representative subset of spectra is already enough to reveal the dominant modifications.

### Detection of candidate modification masses

**Figure 1:**
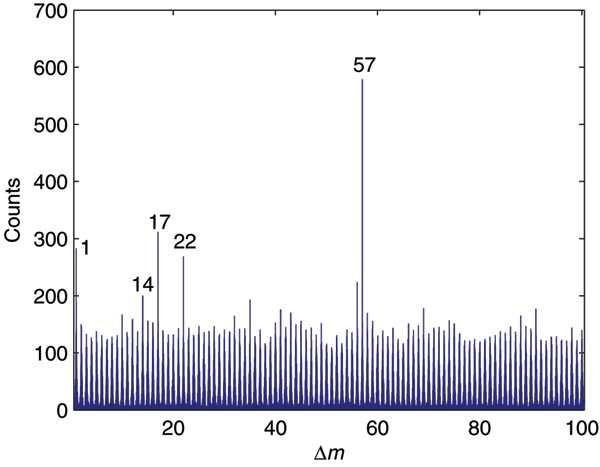
**Δ*m *histogram for data set 1**. Frequent Δ*m *are possibly derived from abundant modifications, because modified and unmodified peptides usually occur simultaneously in the sample.

Given a set of peptide mass spectra, the peptide mass differences (denoted by Δ*m*) between all pairs of spectra are calculated. As an example, Figure 1 illustrates the Δ*m *histogram for data set 1 (described in the Results section). Only those Δ*m *of relatively small values (0–100 Da here) are considered in this paper, since most modifications are small molecules. As stated previously, for abundant modifications, modified and unmodified peptides tend to be present simultaneously in the sample. Therefore, frequent Δ*m *may correspond to possible peptide modifications. Those Δ*m *of significantly high frequency are considered as candidate modification masses, which will be further validated according to their corresponding retention time shifts.

**Figure 2:**
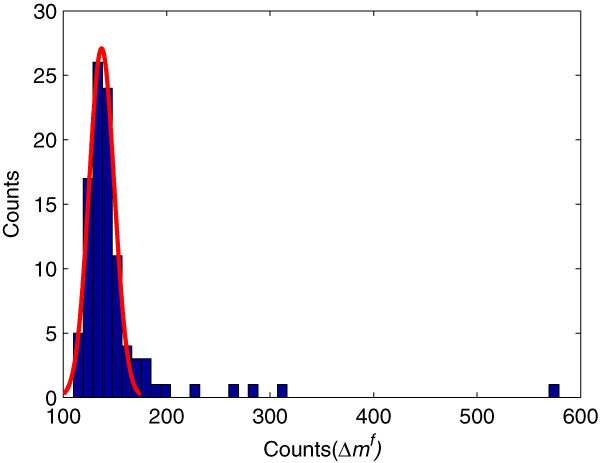
**Frequency Distribution of high-frequency Δ*m *(Δ*m*^*f*^) for data set 1**. The random part of the distribution is very close to a Gaussian distribution, based on which the *p*-value of a $\Delta m_{i}^{f}$ being random can be computed.

To detect statistically significant high-frequency Δ*m*, some probabilistic approach has to be employed. Since peptide masses (and consequently their differences) cluster around unit values, it is inappropriate to assume that all Δ*m *are equally probable. One option is to assume that random Δ*m *follow a mixture of Gaussian distributions centred on unit mass values, and use peptides randomly generated or from some database to generate this mixture distribution. However, it is unknown to what extent this assumption holds and the estimated distribution fits the real spectra data. Instead of using a complicated model, in this paper we resort to a simple yet practically effective approach to the problem posed. For each mass window of one Da around a unit mass value (*i*), the most frequent Δ*m *(denoted by Δ*m*^*f*^) is extracted, that is,

$$\Delta m_{i}^{f}=\underset{\left|\Delta m-i\right|<0.5}{\arg\max}counts\left(\Delta m\right),i=1,2,...,n$$

where *counts*(Δ*m*) is the number of occurrences of Δ*m *and *n *is the number of mass windows. While most $\Delta m_{i}^{f}$ are random events, some may result from peptide modifications. We observe that the frequency, i.e. *counts*(Δ*m*^*f*^), of random Δ*m*^*f*^ approximately follows a Gaussian distribution, as shown in Figure 2. Since it is unknown what type of distribution the frequency of modification-induced Δ*m*^*f*^ comes from, we use a heuristic way to estimate the random part of *counts*(Δ*m*^*f*^). Let *c*_*min *_and *c*_*med *_denote the minimum and the median of *counts*$(\Delta m_{i}^{f})|\begin{array}{l} n\\ i=1 \end{array}$, respectively. Then, those *counts*($\Delta m_{i}^{f}$) smaller than *2c*_*med *_- *c*_*min *_are used to estimate the parameters, i.e., mean and variance, of the random Gaussian distribution of *counts(*Δ*m*^*f*^). Then, for each $\Delta m_{i}^{f}$, a *p*-value is calculated based on this Gaussian distribution. Those $\Delta m_{i}^{f}$ with *p*-values less than a given threshold are considered as candidate modification masses.

### Validation of candidate modification masses

After a list of candidate modification masses are found through the above steps, the next task is to filter out false positives and select a high-confidence subset, since it is possible that the high frequencies of some Δ*m *found in step 1 may be just due to chance rather than real modifications. Given that we do not want to use the fragment peak information (for simplicity and efficiency), it is necessary to look for other type of evidence to verify the candidate modification masses.

In LC-MS/MS experiments, in addition to the peptide masses, the retention time of peptides is another type of information readily available. Since the peptide retention time is orthogonal to the peptide mass, it is an important source of evidence to validate the modifications. The modified and the unmodified forms of a peptide share the same amino acid sequence and differ by a modification group only. As a result, they are similar in physical and chemical properties and thus LC behaviour, i.e. retention time. A modification usually has a relatively small and consistent effect on the retention time of peptides. Therefore, we can expect that if a high-frequency Δ*m *is truly due to a modification, spectral pairs of this Δ*m *will show consistent differences in peptide retention time. For example, a modification may tend to increase or decrease the retention time of peptides to some extent, or simply have no significant influence at all. If a candidate modification mass is accompanied with a wild distribution of retention time differences, this candidate is very likely to be a false alarm.

**Figure 3:**
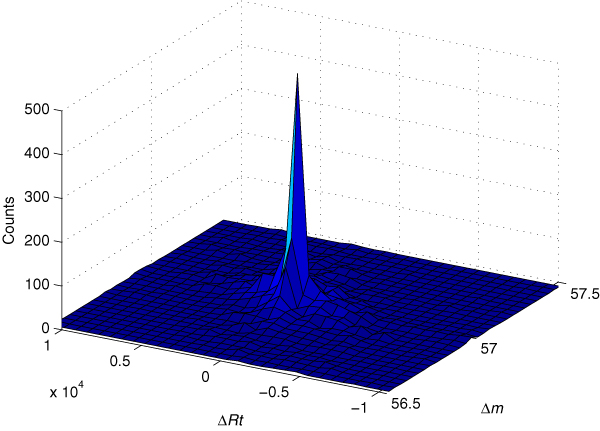
**2-D histogram of Δ*m *and Δ*Rt *for the carbamidomethylation modification found in data set 1**. The sharp peak in both dimensions indicates the actual presence of this modification in the sample, since a modification must cause a fixed change in peptide mass and a slight shift in retention time.

An intuitive way to implement the above analyses is to show the mass differences and the retention time differences (denoted by Δ*Rt*) together in a 2-D histogram. As an example, Figure 3 illustrates a Δ*m *and Δ*Rt *histogram for the carbamidomethylation modification found in data set 1 (see Results section for details). For simplicity, the scan number is used as a substitute for the retention time in this paper. We can see that there is a sharp peak in the histogram, which is produced by the spectral pairs of this modification. As shown, the modification mass is about 57 Da and the modification does not lead to a significant increase or decrease in the peptide retention time. The accurate modification mass and retention time shift can be automatically determined in a statistical framework described below.

### Estimation of mixture distribution

Each pair of spectra is represented by a 2-D vector (denoted by Δ) composed of peptide mass difference Δ*m *and retention time difference Δ*Rt *between the two spectra, i.e.,

Δ = ⟨Δ*m*, Δ*Rt*⟩.

We assume that in the vicinity (e.g. ± 0.5 Da) of each modification mass, Δ follows a bivariate Gaussian mixture distribution with two components, one of which is produced by random spectral pairs and the other by modification-related spectral pairs, or formally,

$$\begin{array}{c} \Delta ∼\alpha_{Rand}G\left(\mu_{Rand},\Sigma_{Rand}\right)+\alpha_{Mod}G\left(\mu_{Mod},\Sigma_{Mod}\right),\\ \alpha_{Rand}+\alpha_{Mod}=1, \end{array}$$

where *G*(*μ*, Σ) is the Gaussian distribution with mean *μ *and covariance matrix Σ, subscripts *Rand *and *Mod *denote random and modification-related spectral pairs, respectively, and *α*_*Rand *_and *α*_*Mod *_are mixing coefficients. The parameters of the mixture distribution can be estimated using the Expectation-Maximization (EM) algorithm.

**Figure 4:**
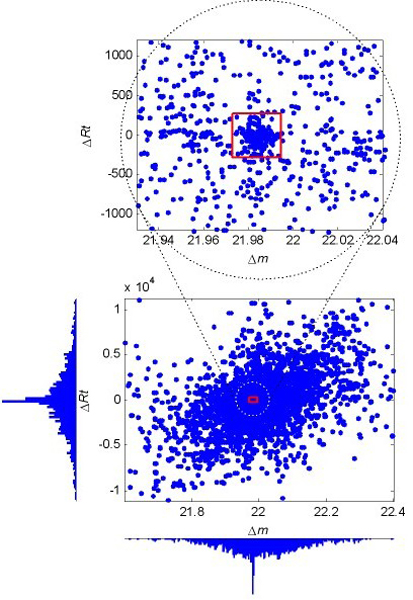
**Scatter-histogram of Δ around the unit mass value 22 for data set 1**. The Δ in the vicinity of each modification mass are assumed to come from a mixture of two Gaussian distributions produced by the random and the modification-related spectral pairs, respectively. The dense data points in the red square, which are automatically localized using the EM algorithm, are mostly derived from modification-related (cation:Na) spectral pairs.

Figure 4 shows the Δ distribution around the unit mass value 22 for data set 1. We can see that the majority of the data follow Gaussian-alike distributions along both dimensions, with a sharp peak in each distribution center. The sharp peaks, which are in fact produced by the cation:Na modification, can also be fitted by Gaussian distributions.

### Detection of spectral pairs

After the modification-related and the random distributions of Δ are estimated in the above step, modification-related spectral pairs can be identified at given posterior error rate. Given an observation of Δ, the posterior probability of the corresponding spectral pair being modification-related is

$$\begin{array}{c} p\left(Mod|\Delta \right)=\frac{p\left(\Delta |Mod\right)P\left(Mod\right)}{p\left(\Delta |Mod\right)P\left(Mod\right)+p\left(\Delta |Rand\right)P\left(Rand\right)}\\ =\frac{f\left(\Delta |\mu_{Mod},\Sigma_{Mod}\right)\alpha_{Mod}}{f\left(\Delta |\mu_{Mod},\Sigma_{Mod}\right)\alpha_{Mod}+f\left(\Delta |\mu_{Rand},\Sigma_{Rand}\right)\alpha_{Rand}} \end{array},$$

where *f *is the Gaussian probability density function:

$$f\left(\Delta |\mu ,\Sigma \right)=\frac{1}{2\pi {\left(\Sigma \right)}^{1/2}}e^{-\frac{1}{2}{\left(\Delta -\mu \right)}^{T}\Sigma^{-1}\left(\Delta -\mu \right)}.$$

Spectral pairs with posterior probability larger than the given threshold are accepted as true modification-related spectral pairs.

### Propagation of peptide assignments

After high-confidence modification-related spectral pairs are found out, the peptide assigned to one spectrum can be propagated to its paired spectrum. It is expected that many spectra that cannot be assigned peptides to via standard database search can be identified in this way. This is especially useful for modification spectra of low signal/noise ratios. For example, according to our observation, cations usually prevent peptides from sufficient fragmentation, resulting in few signal fragment peaks. In addition, some modifications can occur at many sites. For example, according to the annotations in the Unimod database, the carbamidomethylation can possibly occur at the N-term of any peptides and five amino acids anywhere. It is not practical to specify too many variable modifications in current database search engines, which will greatly increase the time of database search and the chance of false positive identifications. Therefore, direct propagation of peptide assignments among paired spectra will be a promising and efficient approach to identifying modified peptides and increasing the interpretation rate of spectra, a major expectation in the proteomics community.

### Inference of modification types

For spectra of high mass accuracy, e.g. spectra produced from FT spectrometers, the Δ*m *values can be resolved in ppm level. Although we used 0.01 Da as the bin width in the Δ*m *histogram, more accurate values of modification masses can be obtained by fitting the 2-D distribution of Δ. Retention time difference Δ*Rt*, as an additional source of information can exclude many random spectral pairs. With accurate modification masses, the types of common modifications can be easily determined by searching modification databases, e.g., Unimod. Retention time shift is another type of evidence to infer modification types. Different modifications have different influences on the retention time of peptides. For example, we observe that oxidation tends to decrease the retention time (by about 700 scans as shown in Table 2). Some rare or unknown modifications may not be registered in databases. In this case, the molecular formula of a modification has to be manually inferred. Special attention should be paid to cation modifications. For an *n*+ cation, the resulting Δ*m *is the atomic mass of this cation minus *n *proton masses. See data sets 1 and 2 in this paper for examples of cation modifications.

## Results and discussion

We used two data sets of mass spectra to test our algorithm. Both data sets were produced from glycoprotein samples. The reason lies in that in order to identify glycosylated peptides, protein samples have to undergo complex treatments, which can possibly introduce chemical modifications. We show that modifications can be efficiently discovered using our algorithm and either including the modifications into the database search process or directly propagating peptides between paired spectra can significantly increase the number of spectra interpreted.

### Data

#### Data set 1

Ig G depleted human plasma from a healthy donor was mixed with WGA, Con A and JAC lectins to enrich most glycoproteins. Then the glycoproteins were reduced, alkylated and digested by trypsin and PNGase F, followed by LC-MS^2 ^analysis. After treating the glycopeptides with PNGase F, the deglycosylated peptides had a +0.984 Da mass drift on asparagine to aspartate. LC-MS^2 ^experiments were performed on an LTQ-FT mass spectrometer. The LTQ-FT mass spectrometer was operated in the data-dependent mode. A full scan survey MS experiment was acquired in the FT-ICR mass spectrometer, and the five most abundant ions detected in the full scan were analyzed by MS^2 ^scan events. This resulted in a total of 8,654 MS^2 ^mass spectra.

#### Data set 2

Ig G depleted human plasma from a healthy donor was mixed with LCH lectins to enrich core fucosylated glycoproteins; then the glycoproteins were reduced, alkylated and digested by trypsin and Endo F3 (treatment with Endo F3 released partial oligosaccharide chain, and leaved the fucosyl GlcNAc reside on the peptides). Samples were separated by SCX and RP HPLC, and then sent to an LTQ-FT mass spectrometer. A full scan survey MS experiment was acquired in the FT-ICR mass spectrometer, and the five most abundant ions detected in the full scan were analyzed by MS^2 ^scan events. An MS^3 ^spectrum was automatically collected when one of the top three intense peaks from the MS^2 ^spectrum corresponded to a neutral loss event of 73.0290 m/z, 48.6860 m/z and 36.5145 m/z. Among the resulted MS^3 ^mass spectra, those that were sure to be from core fucosylated glycopeptides were selected out intelligently in a post-processing step [29]. The final data set consists of a total of 1,528 MS^3 ^mass spectra considered to have been generated from core fucosylated glycopeptides.

### Discovered modifications and spectral pairs

Tables 1 and 2 list the modifications and the number of related spectral pairs discovered in data sets 1 and 2, respectively. The modification masses and the retention time (scan number) shift caused by modifications are automatically learned using the algorithm presented above. The number of spectral pairs related by each modification with posterior probabilities above 0.98 is also given. It is shown that the modification mass values determined from data are very accurate, up to the level of 0.001 Da or even 0.0001 Da. This high mass accuracy is largely due to the use of retention time as a second dimension for detecting spectra pairs. Using peptide masses alone would result in a much lower mass accuracy, e.g. 0.01 Da or so. With a high mass accuracy, the modification types can be easily identified by searching the Unimod modification database. In addition, since the peptide fragmentation data is not used in our algorithm, the computational process is very fast. Except the time for loading mass spectra data from disk, only a couple of minutes are needed.

Among the discovered modifications, some are common in LC-MS/MS experiments, e.g., oxidation, carbamidomethylation and water-/ammonia-loss. Carbamidomethylation is usually specified as a fixed modification on cysteine as an artifact for database search. However, we show here that carbamidomethylation may occur on more specific sites abundantly in a variable manner. Modifications of important biological functions are also found. Methylation is an *in vivo *post-translational modification, while deamidation can occur both *in vivo *and *in vitro*. Other modifications may have been introduced by special sample treatments or from samples themselves, e.g., the sodium cation coming from digestion buffer of glycosidase or lectin binding buffer commonly used in the glycoproteomics research and the iron cation coming from hematoglobins in plasma.

Two types of cation modifications are found. They are the sodium cation (cation:Na) in data set 1 and the very abundant 2+ iron cation (cation:Fe(II)) in data set 2. The Fe(II) modification (about 54 Da) is not registered in the Unimod database and has not been reported before. Note that the Fe(III) modification (about 53 Da) has been found and reported [22,30]. Three evidences make us very confident of the identity of the Fe(II) modification. First, the empirical mass is very close to the theoretical one – a difference of only 0.0016 Da. Second, the retention time shift (Δ*Rt*) caused by this modification is very small, just like other cations, e.g. sodium. Third, we observe that a large proportion of spectra with this modification have higher charge states than their paired spectra without this modification. This can be explained by a polyvalent-cation modification.

### Improvements on peptide identification

There are two ways to make use of the found modification types to improve peptide identification. The most common way is to include them as variable modifications into the database search process. The other is to directly propagate peptide between paired spectra without re-searching the database. Both experiments are conducted. Tables 3 and 4 give the peptide identification results on data sets 1 and 2, respectively. Database search is performed using the pFind search engine [7,31] and the target-decoy database method [32] is used to estimate the FDR of search results. For data set 1, we include the deamidation (at N) introduced by sample preparation and the common oxidation (at M) as variable modifications in the first round of database search. Then, the most abundant carbamidomethylation (at N-term and K) is added in the second round of database search. As a result, 301 more spectra are interpreted, although the increase in the number of peptide identifications is trivial. Based on the first-round database search results, peptide propagation is carried out. It turns out that 537 spectra with the carbamidomethylation modification are assigned with peptides at the same FDR (2%) as database search (we use the maximum posterior error probability as a conservative estimate of FDR, which in theory is larger than actual FDR). Moreover, after peptide propagation between spectra paired by the cation:Na modification, 134 more spectra are assigned with peptides. On data set 2, inclusion of oxidation into the database search increases the number of interpreted spectra by 22, while peptide propagation between spectral pairs related by the cation:Fe(II), oxidation and deamidation increases the number of interpreted spectra by 143, 33 and 19, respectively. Note that cation modifications, e.g. Na and Fe adduct cannot be added into database search as variable modifications. This is because it is not clearly known which amino acids the cations are attached to and the spectra with cation modifications usually have very low signal/noise ratio according to our observation. On average, an increase of 10% in spectra interpretation rate is obtained on these two data sets after considering the discovered modifications.

To sum up, experimental results demonstrate that including the detected modification types into database search can increase the number of interpreted spectra to some extent, while direct peptide propagation between spectral pairs related by modifications can interpret even more spectra. Moreover, it seems that spectra with cation modifications can only be effectively assigned with peptides by peptide propagation.

## Conclusion

In this paper, we have presented a method for detecting abundant modifications in peptide mass spectra using the peptide mass and retention time differences. The method is more accurate than those using peptide masses only and is more efficient than those using peptide fragmentation data. One disadvantage of our method is that it cannot detect low-concentration modifications. But we argue that this method is not designed for this purpose. By using a statistical framework, very accurate modification masses can be obtained and the influence of modifications on the peptide retention time can be quantified. Moreover, modification-related spectral pairs can be detected at a given posterior error rate. An interesting phenomenon observed in this paper is that propagating peptides between paired spectra can interpret even more spectra of modified peptides than including the discovered modifications into database search. Peptide propagation is particularly useful for identifying peptides of cation modifications. In the future, we wish to test the method on more proteomic data sets and incorporate more information to cluster spectra.

## Competing interests

The authors declare that they have no competing interests.

## Authors' contributions

YF conceived and implemented the algorithm, performed data analysis, and wrote the manuscript. WJ and ZL carried out the LC-MS/MS experiments, helped identify the modification types, and participated in writing the manuscript. HW participated in the design of the algorithm and carried out selecting of core fucosylated spectra. ZY, HC, YL, LX, WW, CL and LW provided the support of pFind-series software tools. LX wrote the program for peptide propagation. RS participated in writing the manuscript. WG, HQ and SH supervised the project and revised the manuscript. All authors read and approved the final manuscript.

**Table 1:** Modifications and spectral pairs found in data set 1

**Δ*m *(Da)**		**Δ*Rt *(Scans)**		# of spectral pairs (*pp *> 0.98)*	**Modification type**	**Theoretical mass (Da)**	**Unimod AC #**
				
**Mean**	± 3**Sigma**	**Mean**	± 3**Sigma**				
57.0221	± 0.0186	-31.4	± 387.1	2303	Carbamidomethylation	57.0215	4
21.9839	± 0.0108	-10.5	± 276.2	319	Cation:Na	21.9819	30
17.0254	± 0.0201	-353. 2	± 1360.0	0	Ammonia-loss/Gln → pyro-Glu	17.0265	385/28
14.0157	± 0.0178	-677.6	± 1012.6	0	Methylation/Asp → Glu	14.0157	34/558
0.98943	± 0.0603	176.6	± 1152.6	0	Amidation/Deamidation	0.98402	2/7

*pp stands for the posterior probability of being modification-related. 3Sigma stands for three times of standard deviation. For the last three modifications, spectral pairs of *pp *> 0.98 are not detected out. This is due to the less abundance of these modifications. However, this does not prevent the algorithm from accurately estimating their masses.

**Table 2:** Modifications and spectral pairs found in data set 2.

**Δ*m***		**Δ*Rt***		**# of spectral pairs (*pp *> 0.98)**	**Modification type**	**Theoretical mass**	**Unimod AC #**
				
**Mean**	± 3**Sigma**	**Mean**	± 3**Sigma**				
53.9177	± 0.0180	-15.3	± 222.3	5236	Cation:Fe(II)	53.9193	None
15.9942	± 0.0090	-685.3	± 762.3	4613	Oxidation	15.9949	35
0.9959	± 0.0395	47.4	± 254.9	305	Amidation/Deamidation	0.9840	2/7
18.01	N/A	N/A	N/A	N/A	Water-loss/Glu → pyro-Glu*	18.0106	23/27
57.02	N/A	N/A	N/A	N/A	Carbamidomethylation*	57.0215	4

*Those modifications are found from the 1-D Δ*m *****histogram and manually validated by 2-D <Δm, Δ*Rt*> histogram, but the data points for them are too few to effectively estimate their Δ*m***** and Δ*Rt***** distributions.

**Table 3:** Peptide identification and propagation results on data set 1 (FDR < 2%)

	**Variable modifications**	**# of peptides identified**		**# of spectra interpreted**
			
		**All**	**Glycosylated**	
DB search	Deamidation (N)	479	79	3514
	Oxidation (M)			
	
	Deamidation (N)	484	82	3815
	Oxidation (M)			
	Carbamidomethylation (N-term)			
	Carbamidomethylation (K)			

Peptide propagation*	Carbamidomethylation	-	-	+537
	
	Cation:Na	-	-	+134

*Peptide propagation is conducted based on the peptide identification results of the first-round database search (first row in the table body). Peptide propagation cannot identify more peptides, but significantly increases the number of interpreted spectra.

**Table 4:** Peptide identification and propagation results on data set 2 (FDR < 2%)

	**Variable modifications**	**# of peptides identified**		**# of spectra interpreted**
			
		**All**	**Core fucosylated**	
DB search	Glycosylation (N)	73	73	425
	
	Glycosylation (N)	71	71	447
	Oxidation (M)			

Peptide propagation	Cation:Fe(II)	-	-	+143
	
	Oxidation	-	-	+33
	
	Deamidation	-	-	+19
